# Gestational Age-Specific Complete Blood Count Signatures in Necrotizing Enterocolitis

**DOI:** 10.3389/fped.2021.604899

**Published:** 2021-02-26

**Authors:** Julia M. Pantalone, Silvia Liu, Oluwabunmi O. Olaloye, Erica C. Prochaska, Toby Yanowitz, Melissa M. Riley, Justin R. Buland, Beverly S. Brozanski, Misty Good, Liza Konnikova

**Affiliations:** ^1^University of Pittsburgh School of Medicine, Pittsburgh, PA, United States; ^2^Department of Pathology, University of Pittsburgh, Pittsburgh, PA, United States; ^3^Department of Pediatrics, University of Pittsburgh, Pittsburgh, PA, United States; ^4^Department of Pediatrics, Yale School of Medicine, New Haven, CT, United States; ^5^Division of Infectious Diseases, Department of Pediatrics, Johns Hopkins University School of Medicine, Baltimore, MD, United States; ^6^Department of Pediatrics, Washington University School of Medicine, St. Louis, MO, United States; ^7^Department of Immunology, University of Pittsburgh, Pittsburgh, PA, United States; ^8^Department of Developmental Biology, University of Pittsburgh, Pittsburgh, PA, United States

**Keywords:** immunology, complete blood count, gestational age, necrotizing entercolitis, pre-maturity

## Abstract

**Objective:** Necrotizing enterocolitis (NEC) is characterized by peripheral cell abnormalities, yet few studies have analyzed the complete blood count (CBC) specifically by gestational age (GA). Our objective was to describe GA-specific immune abnormalities in NEC through a comprehensive analysis of the CBC differential.

**Methods:** Using a cohort of 246 infants (177 cases, 69 controls) admitted to neonatal intensive care units at a single institution, we retrospectively analyzed CBCs around illness onset in NEC cases compared with controls. Cases included surgical NEC (S-NEC, 34.5%) and medical NEC (M-NEC, 65.5%). Infants were divided into those born at GA <33 and ≥33 weeks. Differences in CBC values were described as absolute and percent changes at NEC onset from baseline and at antibiotic completion after NEC. We used machine learning algorithms based on the CBC at NEC to generate predictive models for diagnosis.

**Results:** At NEC onset, there was an acute drop in monocytes and lymphocytes along with a rise in bands in S-NEC infants born <33 weeks compared with M-NEC. In comparison, both M-NEC and S-NEC ≥33 weeks had a percent drop in neutrophils at diagnosis compared with controls. At antibiotic completion, monocytes in S-NEC <33 weeks significantly rose compared with M-NEC, yet for S-NEC ≥33 weeks, bands significantly dropped compared with M-NEC. Predictive modeling was able to accurately predict S-NEC from M-NEC and controls.

**Conclusion:** There are discrete leukocyte patterns in NEC based on GA. The CBC at diagnosis may be useful in identifying patients who will require surgery.

## Introduction

Necrotizing enterocolitis (NEC) is a neonatal gastrointestinal emergency with significant morbidity and mortality (1). Although the pathophysiology of NEC remains incompletely understood, the underlying mechanisms are likely multifactorial and involve immune dysregulation, dysbiosis, and intestinal injury. Clinical data suggest that these mechanisms may differ between early pre-term and late pre-term/term infants (2). Indeed, NEC most likely encompasses a spectrum of different pathologies that present similarly and must be contextualized based on infants' individual risk factors (2, 3).

Circulating immune cells likely contribute to illness progression and may be helpful biomarkers in diagnosing infants suspected of having NEC and/or stratifying disease severity. Prior studies have shown that an acute drop from baseline in monocyte count is suggestive of NEC diagnosis (4, 5). Similarly, neutropenia at illness onset is seen in severe NEC and is associated with greater extent of disease and surgical intervention (6–8). A number of studies have suggested that alterations in lymphocytes are associated with NEC, although the data are mixed (9–11). Some studies report that lymphocytosis is associated with worse outcomes (9, 10), while others show that lymphopenia is correlated with mortality in NEC (11).

Both gestational age (GA) and chronological age are likely important factors that contribute to the complete blood count (CBC). In neonates, absolute cell counts vary based on gestational and postnatal ages and likely have different clinical implications in light of an infant's pre-maturity (12–14). For instance, a previous study that examined the lymphocyte count from a CBC drawn on the day of NEC diagnosis showed paradoxical findings based on GA (9). There are limited studies evaluating CBCs in infants with NEC based on GA and few studies that look at changes in peripheral counts during recovery from NEC. Here, we aimed to analyze serial CBCs obtained around NEC diagnosis in a cohort of infants stratified by GA, specifically evaluating changes in peripheral cell counts in reference to normalized baseline values in an attempt to adjust for these age-related variations. Through this comprehensive evaluation of the CBC differential, we identified two discrete peripheral immune signatures in NEC based on GA.

## Methods

### Patient Enrollment

The study was approved by the University of Pittsburgh Institutional Review Board (IRB PRO09110437). In this case–control study, infants admitted to two neonatal intensive care units (level III, delivery hospital and level IV, tertiary referral center) at an academic health care system were prospectively enrolled from 2010 to 2019, and the data were analyzed retrospectively. Parents of infants were approached after NEC diagnosis and were enrolled upon consent. GA-matched infants without NEC born within the same calendar year served as controls. All infants were analyzed together and then stratified into two cohorts based on GA at birth <33 and ≥33 weeks. This cutoff was chosen due to the known GA distribution of NEC, in that most cases of NEC (>77%) occur in infants <32 or 33 weeks gestation while relatively fewer cases occur in infants closer to term (15). GA was determined by obstetrical assessment. Diagnosis of NEC (modified Bell stage II or greater) was performed by neonatologists based on clinical symptoms (e.g., bloody stools, abdominal distension), abdominal radiographs, and surgical tissue specimens. Infants diagnosed with NEC were categorized into treatment groups: medical NEC (M-NEC) were cases that received only medical treatment including *nil per os*, parenteral nutrition, and antibiotics; surgical NEC (S-NEC) included cases that received any surgical intervention (e.g., peritoneal drainage, bowel resection) during their initial treatment. NEC was differentiated from spontaneous intestinal perforation (SIP) by timing of intestinal perforations. Infants with SIP (*n* = 2) or those concerning for SIP–NEC overlap were excluded from the study (*n* = 1). If infants had recurrence of NEC during their hospitalization, only data from the first episode was analyzed. Controls were identified by a clinical research coordinator to match NEC infants within 2 days of birth GA and included upon parental consent. Controls included healthy infants (*n* = 26), infants with other pediatric gastrointestinal diseases (*n* = 3), and infants with evaluations for sepsis (early-onset sepsis, *n* = 22; late onset sepsis, *n* = 13), or NEC (modified Bell stage I, *n* = 5), but excluded any infant with a confirmed infection by culture-positive microbiology data (*n* = 7).

### Patient Demographics and Data Collection

**Figure 1:**
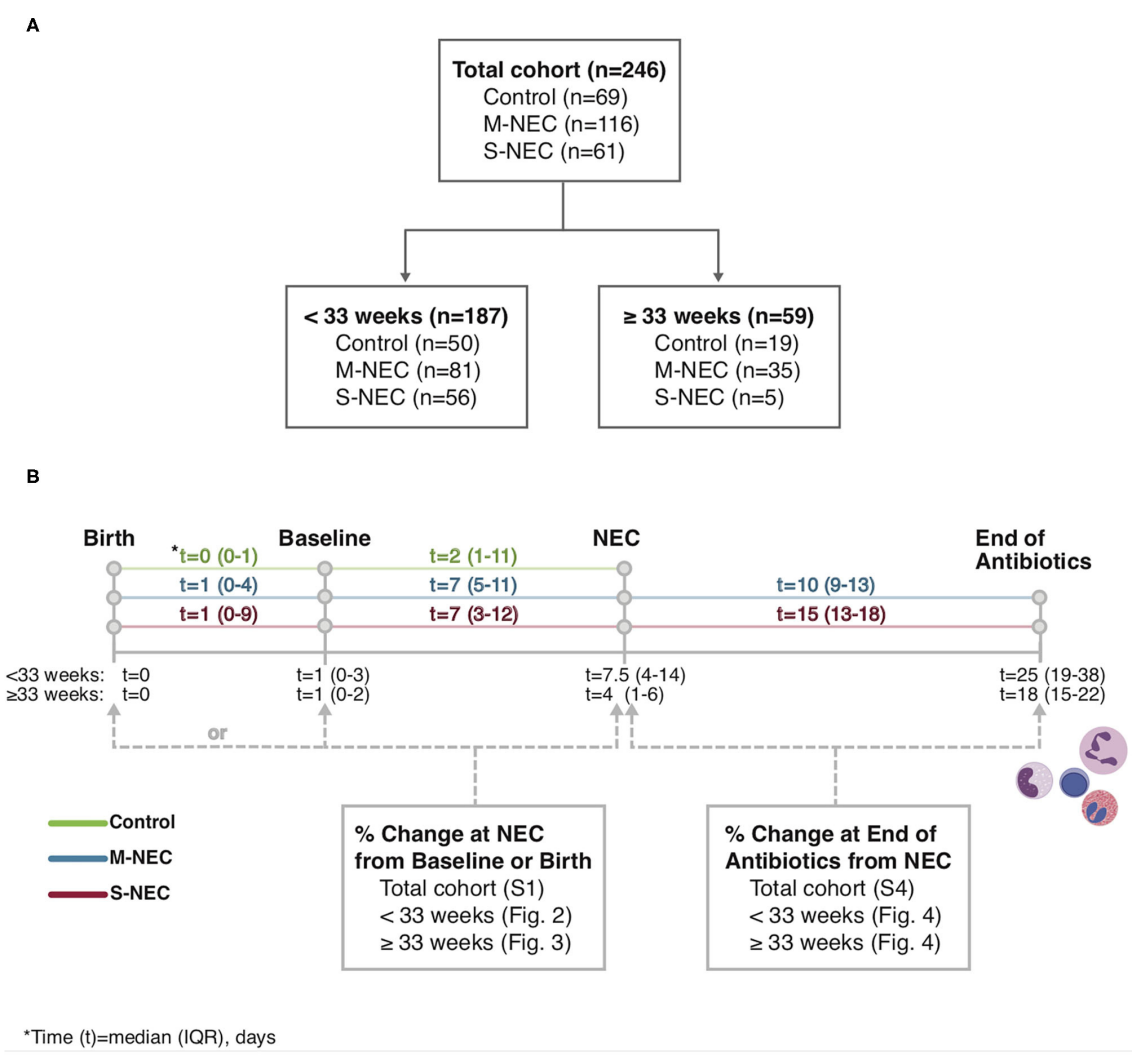
Study flowchart **(A)** and timeline **(B)**. Complete blood count (CBC) values obtained at birth, baseline, necrotizing enterocolitis (NEC) diagnosis, and the end of the antibiotic course were recorded from the electronic medical record.

Demographic and relevant clinical information were recorded, including sex, GA, birth weight (BW), comorbidities, small for gestational age (SGA, <10% weight for GA), time to NEC diagnosis, blood culture results, and antibiotic duration. Data retrieved from CBC differentials included white blood cell (WBC) count, absolute monocyte count (AMC), absolute lymphocyte count (ALC), absolute eosinophil count (AEC), absolute neutrophil count (ANC), and absolute band count. These data were recorded from CBCs obtained at birth, at a baseline timepoint, at time of NEC diagnosis, and within 3 days of antibiotic course completion (Figure 1). Baseline was defined as the last available CBC recorded before NEC onset, including those obtained at birth. The CBC used at “NEC diagnosis” for control infants was the CBC obtained for any clinically indicated blood draw after birth. For comparisons at illness onset, absolute cell counts were analyzed as percent change at NEC diagnosis compared with those at baseline or birth. For comparisons at the end of antibiotics, absolute cell counts were analyzed as percent change at antibiotic completion compared with those obtained at NEC diagnosis.

### Statistical Analysis

Statistical analysis was performed using SPSS version 26.0 (IBM SPSS Statistics, Armonk, NY) and graphed with GraphPad Prism version 8 (GraphPad Software, San Diego, CA). Categorical data were summarized with proportions of patients and compared using Fischer's exact-test. Continuous variables were expressed as medians (interquartile ranges, IQRs) and visualized as box-and-whisker plots. When continuous variables were normally distributed, *t*-tests and ANOVA were used. Continuous and non-normally distributed data were analyzed using Mann–Whitney and Kruskal–Wallis-tests. *Post-hoc* analysis was performed using Bonferroni correction for multiple tests between S-NEC, M-NEC, and control groups. Significance was defined as *p*-value < 0.05.

### Machine Learning Prediction Model

For the entire cohort, we used machine learning algorithms to generate predictive models to distinguish control and NEC subjects based on CBC findings while also accounting for gestational and postnatal age differences. In the modeling, 246 patients were included with GA and postnatal age at NEC diagnosis as continuous variables, along with 12 features obtained at NEC diagnosis or the equivalent timepoint in controls: WBC, AMC, ALC, AEC, ANC, and absolute bands at NEC diagnosis; and WBC, AMC, ALC, AEC, ANC, and absolute bands percent change from baseline at diagnosis. Patients with more than half missing values across these 12 features were removed and the other missing values were imputed by the k-nearest neighbor method (16, 17). Data on 234 patients within three NEC categories were used for further analysis (58 control, 116 M-NEC, and 60 S-NEC). The t-stochastic neighbor embedding (t-SNE) (18) method was applied to reduce and visualize the data in two dimensions. A 10-fold cross-validation (CV) method was employed to evaluate the model's prediction ability. That is, the 234 individuals were randomly split into 10 groups; for each round, 9-folds of the data were used as training set to build up the classification model, and the model was applied to the remaining one-fold for testing. We repeated this process 10 times until all subjects were predicted for the evaluation. To distinguish the categories, three machine learning algorithms were employed for multi-outcome prediction: linear discriminant analysis (LDA) (19), support vector machine (SVM) (20, 21), and random forest (RF) (22). Overall accuracy and adjusted Rand index (ARI) (23) were used as prediction evaluation measurements.

A similar 10-fold CV method was applied for binary outcome to compare S-NEC with controls and S-NEC with M-NEC. We employed LDA, SVM, RF, and logistic regression models for the comparison and applied accuracy, sensitivity, specificity, and Youden index (sensitivity + specificity – 1) for overall evaluation of the models. Best models were selected by 10-fold CV. To evaluate the importance of the 12 features when comparing S-NEC with M-NEC and control groups, we used the whole cohort to rank the importance of each feature based on their RF impurity measurement and a binary decision tree (24).

## Results

There were 246 infants included in the study: 177 with NEC and 69 controls. Of infants with NEC, 116 (65.5%) had M-NEC and 61 (34.5%) had S-NEC. Demographic and clinical characteristics are described in Table 1. The majority of infants in our cohort (187, 76%) were born <33 weeks. S-NEC cases were significantly more pre-mature (median GA 28 + 0 week, IQR 26 + 1–30 + 2) and smaller (median BW 960 g, IQR 724–1,320) than both M-NEC cases (GA 31 + 4 weeks, 29 + 3–33 + 3; BW 1,552 g, 1,154–1,974) and control infants (GA 31 + 3 weeks, 28 + 6–33 + 3; BW 1,467 g, 1,213–1,886). Similarly, S-NEC cases were more likely to have other comorbidities, including patent ductus arteriosus and intraventricular hemorrhage, compared with M-NEC and controls. When restricted to infants born <33 weeks gestation, S-NEC remained more pre-mature and smaller at birth (Supplementary Table 1). Infants ≥33 weeks were more likely to be SGA (12/59, 20%) compared with those born <33 weeks (12/187, 6.4%, *p* = 0.003).

**Table 1:** Patient demographics and clinical characteristics.

	**Total cohort (*****n*** **=** **246)**			
	**Medical NEC (*n* = 116)**	**Surgical NEC (*n* = 61)**	**Controls (*n* = 69)**	***p*-value**
**Gestational age**				
Median (IQR), weeks + days	31 + 4 (29 + 3 to 33 + 3)	28 + 0 (26 + 1 to 30 + 2)	31 + 3 (28 + 6 to 33 + 3)	<0.001^a^
≥33 weeks, no. (%)	35 (30.2%)	5 (8.2%)	19 (28%)	0.004^b^
<33 weeks, no. (%)	81 (69.8%)	56 (92%)	50 (72%)	
**Birth weight**				
Median (IQR), g	1,552 (1,154 to 1,974)	960 (724 to 1,320)	1,467 (1,213 to 1,886)	<0.001^a^
>1,500 g, no. (%)	63 (54.3%)	12 (20%)	33 (48%)	<0.001^b^
<1,500 g, no. (%)	53 (45.7%)	49 (80%)	36 (52%)	
**Female**, no. (%)	51 (44.0%)	19 (31%)	34 (49%)	0.10^b^
**Comorbidities**				
CLD, no. (%)	20 (17.2%)	28 (46%)	7 (10%)	<0.001^b^
IVH, no. (%)	27 (23.3%)	22 (36%)	10 (14%)	0.02^b^
PDA, no. (%)	17 (14.7%)	23 (38%)	7 (10%)	<0.001^b^
ROP, no. (%)	28 (24.1%)	23 (38%)	6 (8.7%)	<0.001^b^
SGA, no. (%)	13 (11.2%)	7 (11%)	4 (5.8%)	0.43^b^
**Died**, no. (%)	1 (0.9%)	12 (20%)	1 (1.4%)	<0.001^b^
**Postnatal age at NEC onset**, median (IQR), days	10 (6 to 18)	11 (6 to 24)	4 (1 to 15)	<0.001^a^
**Postnatal age at baseline**, median (IQR), days	1 (0 to 4)	1 (0 to 9)	0 (0 to 1)	<0.001^a^
**Time between baseline and NEC onset**, median (IQR), days	7 (5 to 11)	7 (3 to 12)	2 (1 to 11)	0.001^a^
**Postnatal age at end of antibiotics**, median (IQR), days	22 (16 to 28)	29 (20 to 41)	–^d^	<0.001^c^
**Antibiotic duration**, median (IQR), days	10 (9 to 13)	15 (13 to 18)	–^d^	<0.001^c^
**Positive blood culture at NEC diagnosis**, no. (%)	2 (1.7%)	13 (21%)	–^d^	<0.001^b^

*NEC, necrotizing enterocolitis; CLD, chronic lung disease; IVH, intraventricular hemorrhage; PDA, patent ductus arteriosus; ROP, retinopathy of pre-maturity; SGA, small for gestational age; g, grams; IQR, interquartile range*.

a *Kruskal–Wallis-test*.

b *Fischer exact-test*.

c *Mann–Whitney*.

d *Not applicable to control group*.

For all comparisons, CBCs at the time of NEC diagnosis for patients with NEC were compared with CBCs obtained for other reasons in controls. For brevity, this timepoint is referred to as “NEC diagnosis” for both cases and controls throughout the text. In the entire cohort, CBC values were available for 234 infants at baseline and 235 infants at NEC diagnosis. Of NEC cases, 93/177 (52.5%) infants had a CBC at antibiotic course completion. Baseline CBCs were obtained at a median of 7 days prior to NEC diagnosis (range 1–38 days) for cases and 2 days (range 0–76 days) for controls (Figure 1). In the entire cohort, controls had significantly younger postnatal ages (median 4 days, IQR 1–15) at their comparison NEC timepoint compared with both M-NEC cases (10 days, 6–18, *p* < 0.001) and S-NEC cases (11 days, 6–24, *p* < 0.001). This difference in postnatal age at NEC onset was also seen when the cohort was restricted to infants <33 weeks and infants ≥33 weeks (Supplementary Table 1). For S-NEC cases, the CBCs at illness onset were obtained a median (IQR) of 1 day (0–2) prior to surgical intervention (data not shown). Median (IQR) antibiotic duration was 10 days (9–13) for M-NEC and 15 days (13–18) for S-NEC.

### A Drop in Lymphocytes and Rise in Bands at Diagnosis Differentiates S-NEC From Controls in the Combined Cohort

We initially analyzed the whole cohort. There were no significant differences in peripheral cell counts at birth or baseline between S-NEC, M-NEC, and controls. However, comparing the CBCs collected at NEC diagnosis with those at baseline revealed a significant absolute and percent drop in ALC (Supplementary Figures 1A,B) and rise in band count (Supplementary Figures 1C,D) for S-NEC compared with controls, along with a significant percent rise in bands for M-NEC compared with controls. Additionally, S-NEC was marked by a lower ALC and AEC, but higher band count at diagnosis compared with M-NEC (Supplementary Figures 1A,C,E). For changes in neutrophil counts, both M-NEC and S-NEC cases had a significant absolute drop in ANC at NEC diagnosis compared with controls (Supplementary Figure 1G). When analyzed as a change from baseline, M-NEC cases had a significant percent drop in ANC at NEC diagnosis compared with controls (Supplementary Figure 1H). No other differences in CBC values were observed (Supplementary Figures 1I–L). Given that NEC appears to be clinically distinct in early pre-term and near-term infants, we then stratified the cohort by GA at birth to investigate GA-related immune distinctions.

### A Decrease in Monocytes and Lymphocytes at Diagnosis Differentiates S-NEC From M-NEC and Controls in Infants <33 Weeks

**Figure 2:**
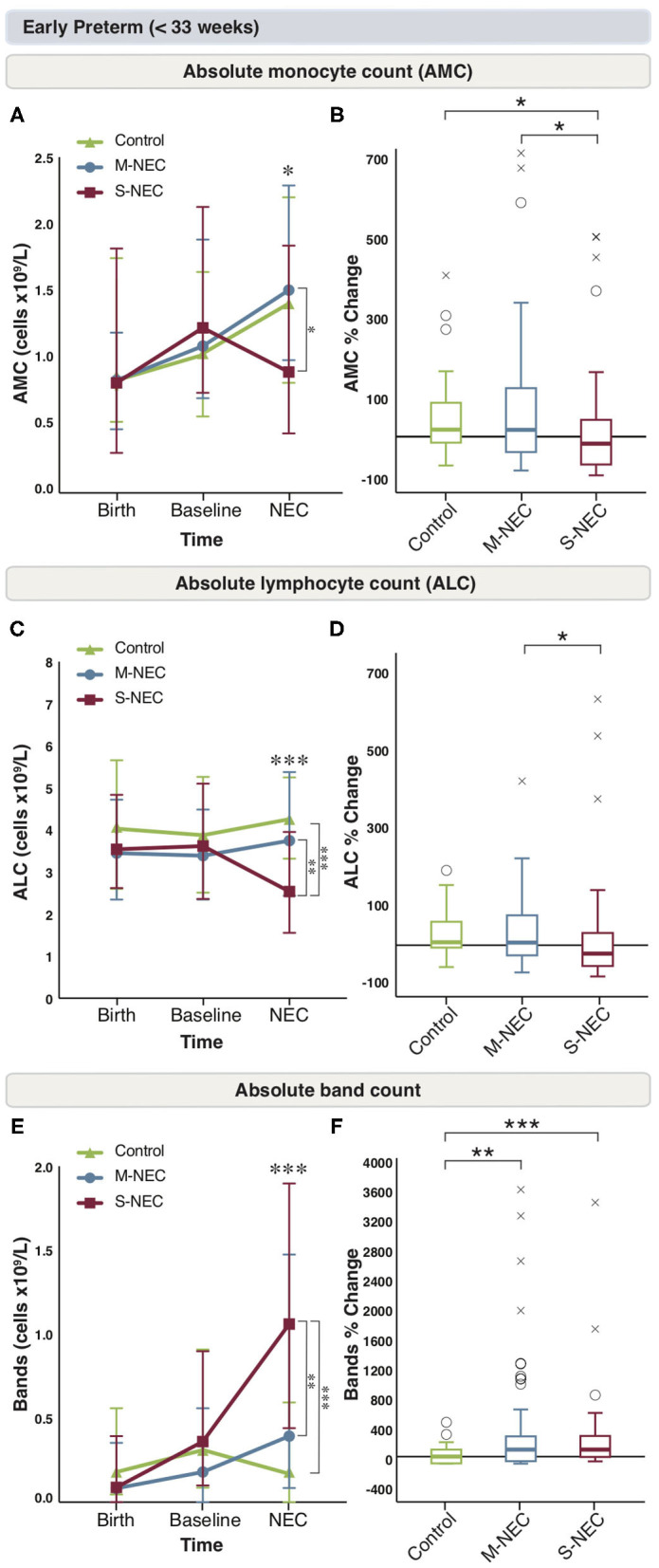
Changes in peripheral cell counts for infants <33 weeks. Median absolute levels of monocytes **(A)**, lymphocytes **(C)**, and bands **(E)** are plotted at birth, baseline, and NEC diagnosis. Tukey boxplots for percent changes in monocytes **(B)**, lymphocytes **(D)**, and bands **(F)** at NEC diagnosis compared with baseline. Bars represent 25 and 75% percentiles **(A,C,E)** or 1.5×the interquartile ranges (IQR) **(B,D,F)**. *Significance for absolute cell count; *p < 0.05, **p < 0.01, ***p < 0.001. *Significance for percent change; *p < 0.05, **p < 0.01, ***p < 0.001. °Indicates outliers (>1.5 × IQR) and ^x^indicates extreme outliers (>3 × IQR).

For infants born <33 weeks, birth and baseline values were similar between all groups (Figure 2 and Supplementary Figure 2). However, at diagnosis, infants with S-NEC had a significant percent drop from baseline in AMC compared with M-NEC and controls (Figures 2A,B). Additionally, S-NEC had lower absolute monocyte levels (median 0.9 × 10^9^/L, IQR 0.45–1.84) than M-NEC cases (1.51 × 10^9^/L, 0.99–2.29, *p* = 0.01) (Figure 2A). When compared with a presymptomatic baseline, monocytes decreased a median (IQR) of 18.01% (−71.83 to 43.83) in S-NEC, compared with a rise in monocytes in M-NEC (17.75%, −39.91 to 125.54, *p* = 0.02) and controls (17.82%, −16.6 to 84.13, *p* = 0.04) (Figure 2B).

Similarly, S-NEC cases had lower absolute lymphocyte levels at NEC onset (2.54 × 10^9^/L, 1.56–3.95) than M-NEC (3.75 × 10^9^/L, 2.44–5.38, *p* = 0.003) and controls (4.26 × 10^9^/L, 3.28–5.23, *p* < 0.001) (Figure 2C). When analyzed as a change from baseline, there was a significant percent drop in ALC at diagnosis in S-NEC (−24.28%, −60.94 to 35.79) compared with M-NEC (7.33%, −29.75 to 86.73, *p* = 0.04), but not compared with controls (9.93%, −11.31 to 66.16, *p* = 0.05) (Figure 2D).

S-NEC cases also had an absolute band count that was nearly three times higher at diagnosis (1.06 × 10^9^/L, 0.44–1.90) than M-NEC (0.39 × 10^9^/L, 0.09–1.48, *p* = 0.005) and controls (0.19 × 10^9^/L, 0–0.61, *p* < 0.001) (Figure 2E). Additionally, there was a significant percent rise in bands at diagnosis from baseline in both S-NEC (100%, −4.1 to 293.23, *p* < 0.001) and M-NEC cases (100%, −66.13 to 288.39, *p* = 0.002) compared with controls (−23.74%, −100 to 77.43) (Figure 2F). Similar to the combined cohort, S-NEC infants born <33 weeks had lower AEC than M-NEC (Supplementary Figure 2E) at illness onset. No other significant differences were observed (Supplementary Figures 2A–F).

### A Drop in Neutrophils Is Seen in NEC Cases ≥33 Weeks

**Figure 3:**
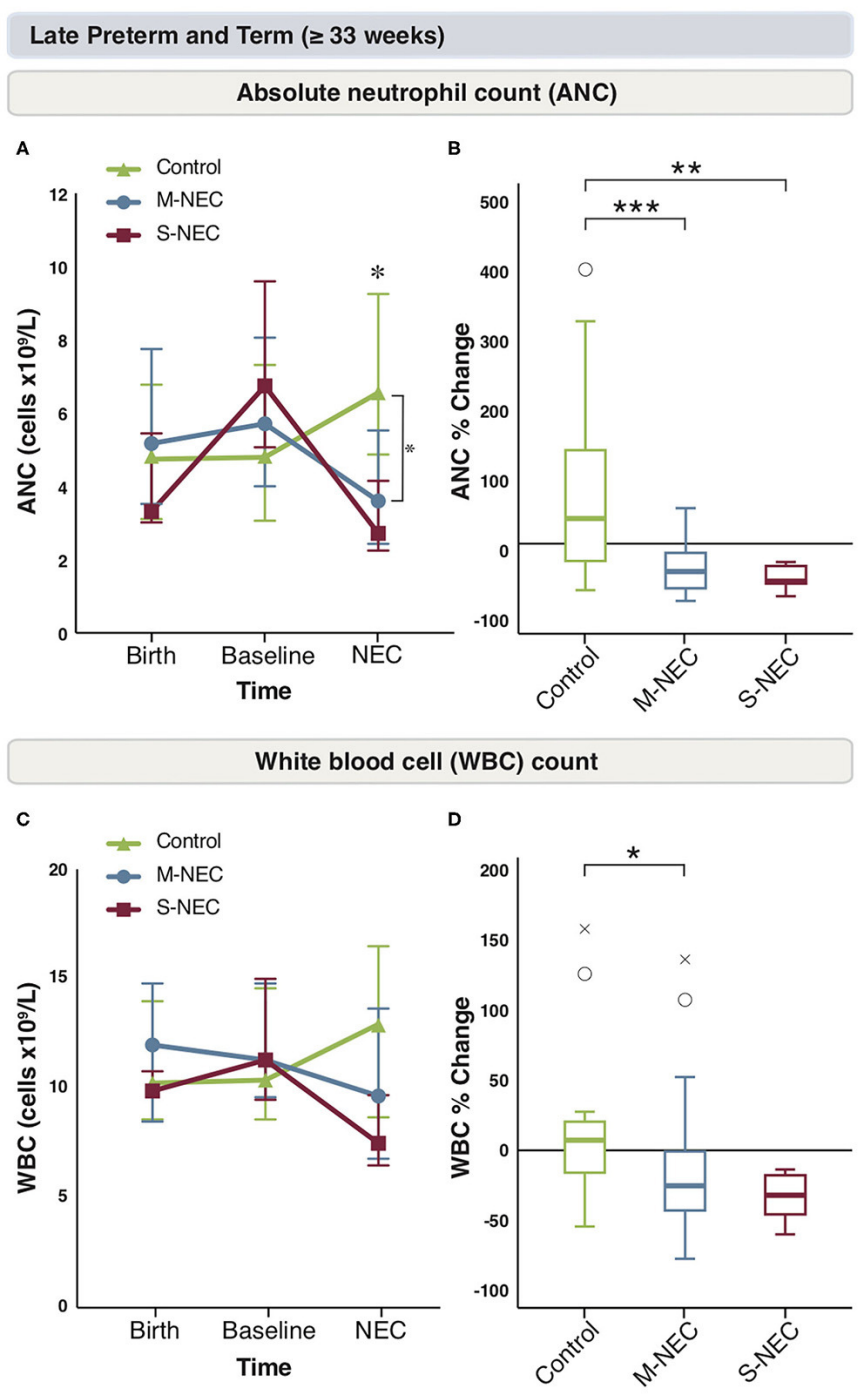
Changes in peripheral cell counts for infants ≥33 weeks. Median absolute levels of neutrophils **(A)** and WBC count **(C)** are plotted at birth, baseline, and NEC diagnosis. Tukey boxplots for percent changes in neutrophils **(B)** and WBC count **(D)** at NEC diagnosis compared with baseline. Bars represent 25 and 75% percentiles **(A,C)** or 1.5× the IQR **(B,D)**. *Significance for absolute cell count; *p < 0.05. *Significance for percent change; *p < 0.05, **p < 0.01, ***p < 0.001. °Indicates outliers (>1.5 × IQR) and ^x^indicates extreme outliers (>3 × IQR).

Again, birth and baseline CBC values were similar between all groups in infants ≥33 weeks (Figure 3 and Supplementary Figure 3). In comparison to the more pre-mature group, for infants born at ≥33 weeks, there were no significant absolute or percent differences in monocytes, lymphocytes, bands, or eosinophils at diagnosis between treatment groups (Supplementary Figures 3A–H). However, similar to the combined cohort, M-NEC cases ≥33 weeks had significantly lower ANC at diagnosis (median 3.60 × 10^9^/L, IQR 2.44–5.54) compared with controls (6.63 × 10^9^/L, 4.9–9.26, *p* = 0.02) with a similar, but not significant, trend in S-NEC cases (2.72 × 10^9^/L, 2.25–4.16, *p* = 0.10) compared with controls (Figure 3A). Furthermore, both S-NEC and M-NEC cases ≥33 weeks had a significant percent drop in ANC at diagnosis from baseline (median −55.62%, IQR −59.01 to −33.17, *p* = 0.01; −40.96%, −66.18 to −13.5, *p* < 0.001, respectively) compared with controls (39.52, −21.36 to 138.62) (Figure 3B).

Similarly, M-NEC cases had a significant percent decrease in WBC count at diagnosis from baseline (−26.19%, −44.57 to −0.86) compared with controls (8.19%, −14.14 to 24.04, *p* = 0.03) (Figures 3C,D).

### Lymphocyte Counts Do Not Fully Recover in S-NEC Cases Born <33 Weeks at the End of Antibiotics

**Figure 4:**
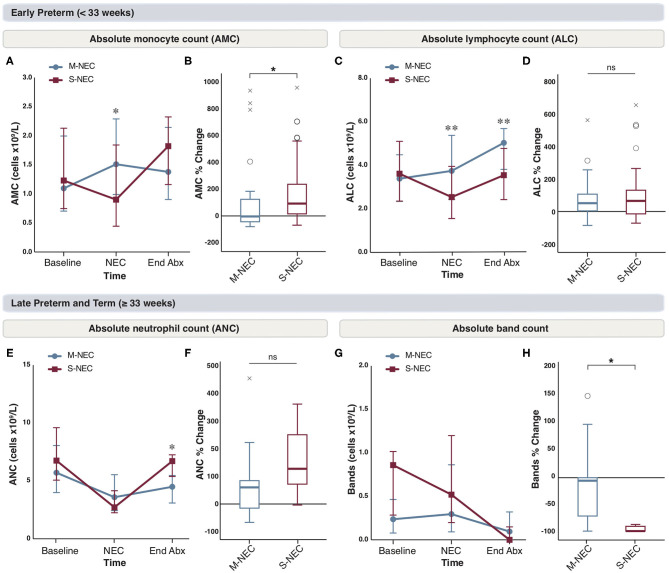
Changes in peripheral cell counts in infants <33 weeks **(A–D)** and ≥33 weeks **(E–H)** at the end of antibiotic treatment. Median absolute levels of monocytes **(A)**, lymphocytes **(C)**, neutrophils **(E)**, and bands **(G)** are plotted at baseline, NEC diagnosis, and the end of the antibiotic course (End Abx). Tukey boxplots for percent changes in monocytes **(B)**, lymphocytes **(D)**, neutrophils **(F)**, and bands **(H)** at end of antibiotics compared with NEC diagnosis. Bars represent 25 and 75% percentiles **(A,C,E,G)** or 1.5× the IQR **(B,D,F,H)**. *Significance for absolute cell count; *p < 0.05, **p < 0.01. *Significance for percent change; *p < 0.05. °Indicates outliers (>1.5 × IQR) and ^x^indicates extreme outliers (>3 × IQR).

We were interested in evaluating the role of antibiotics on circulating immune cells in the recovery period from NEC. CBC components obtained at antibiotic completion were compared with those obtained at illness onset between S-NEC and M-NEC. For infants <33 weeks, the differences observed in AMC at NEC diagnosis were no longer seen after antibiotic treatment, and both groups had similar AMC at the end of antibiotics (Figure 4A). However, when comparing the percent difference in monocyte count before and after antibiotics, S-NEC had a significantly higher rise in AMC (median 92.84%, IQR 15.92 to 237.14) than M-NEC (−3.57%, −43.7 to 124.92, *p* = 0.01) over their treatment course (Figure 4B). Interestingly, M-NEC cases born <33 weeks continued to have higher ALC (5.04 ×10^9^/L, 3.82–5.69) than S-NEC (3.55 ×10^9^/L, 2.43–4.78, *p* = 0.006) even at the end of antibiotics, suggesting an incomplete lymphocyte recovery in S-NEC cases (Figures 4C,D). No other differences were observed in infants <33 weeks (Supplementary Figure 4).

For infants born ≥33 weeks, although there were no differences in absolute neutrophil levels at the time of NEC diagnosis, S-NEC cases had significantly higher ANC at antibiotic completion (6.72 ×10^9^/L, 5.39–7.25) than M-NEC (4.49 ×10^9^/L, 3.1–5.47, *p* = 0.04) (Figure 4E). However, when comparing the percent change in ANC between M-NEC and S-NEC over the course of antibiotics, there was no significant difference between groups (*p* = 0.19) (Figure 4F). Similarly, while M-NEC and S-NEC had similar absolute band counts at NEC diagnosis, S-NEC had a more pronounced percent drop in bands by antibiotic completion (−100%, −100 to −91.03) compared with M-NEC (−5.72%, −71.71 to 0, *p* = 0.02) (Figures 4G,H). No other differences were observed (Supplementary Figure 4).

### Predictive Modeling Using the CBC at NEC Diagnosis Can Distinguish S-NEC From Controls

**Figure 5:**
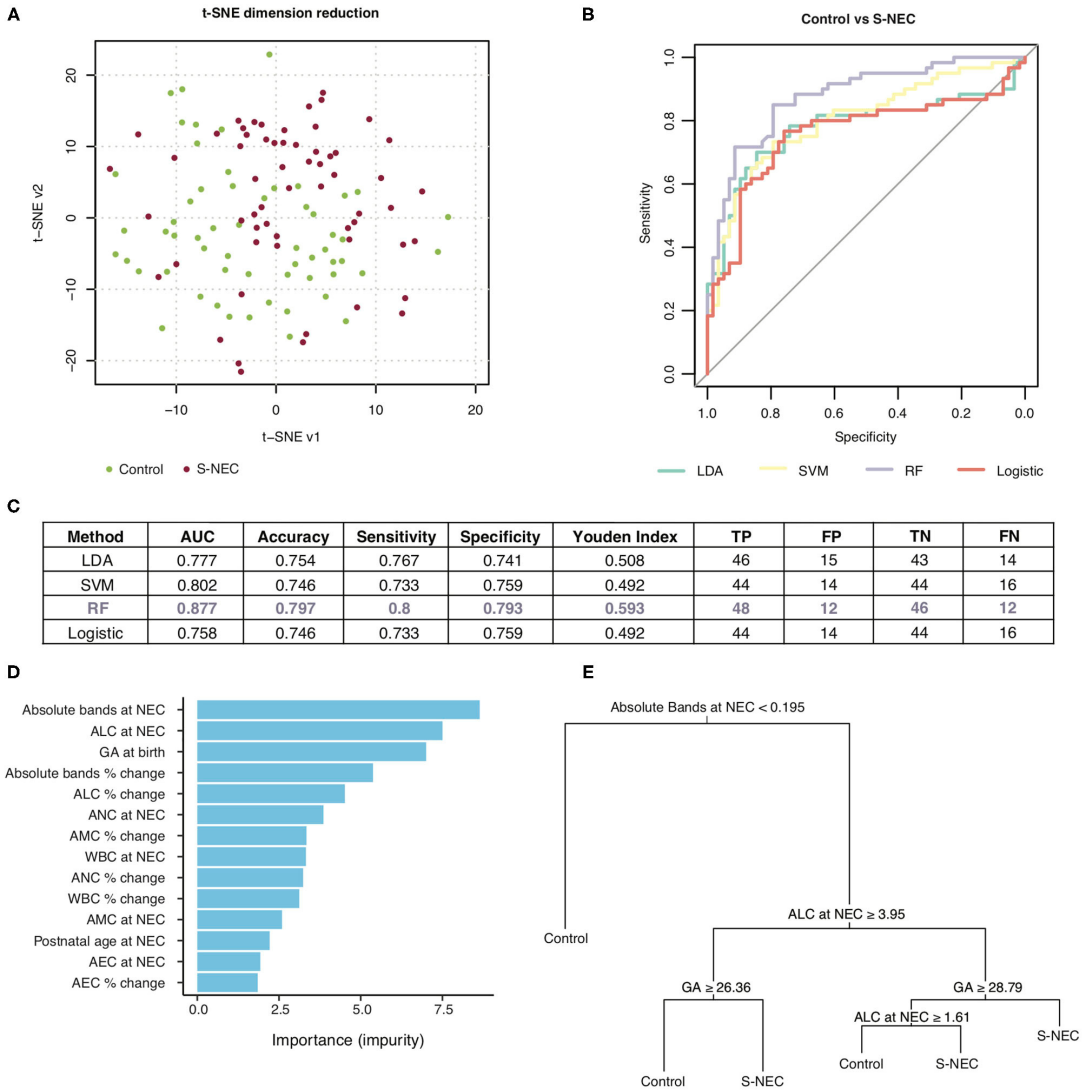
Machine learning prediction model for two-way comparison of surgical NEC (S-NEC) and controls. **(A)** t-SNE separation of S-NEC and controls. **(B)** Receiver-operating characteristic curve for models using LDA, SVM, RF, and logistic regression. **(C)** Table of model characteristics. **(D)** Contribution of each CBC component to the model. **(E)** Representative decision tree. AUC, area under the curve; TP, true positive; FP, false positive; TN, true negative; FN, false negative.

**Figure 6:**
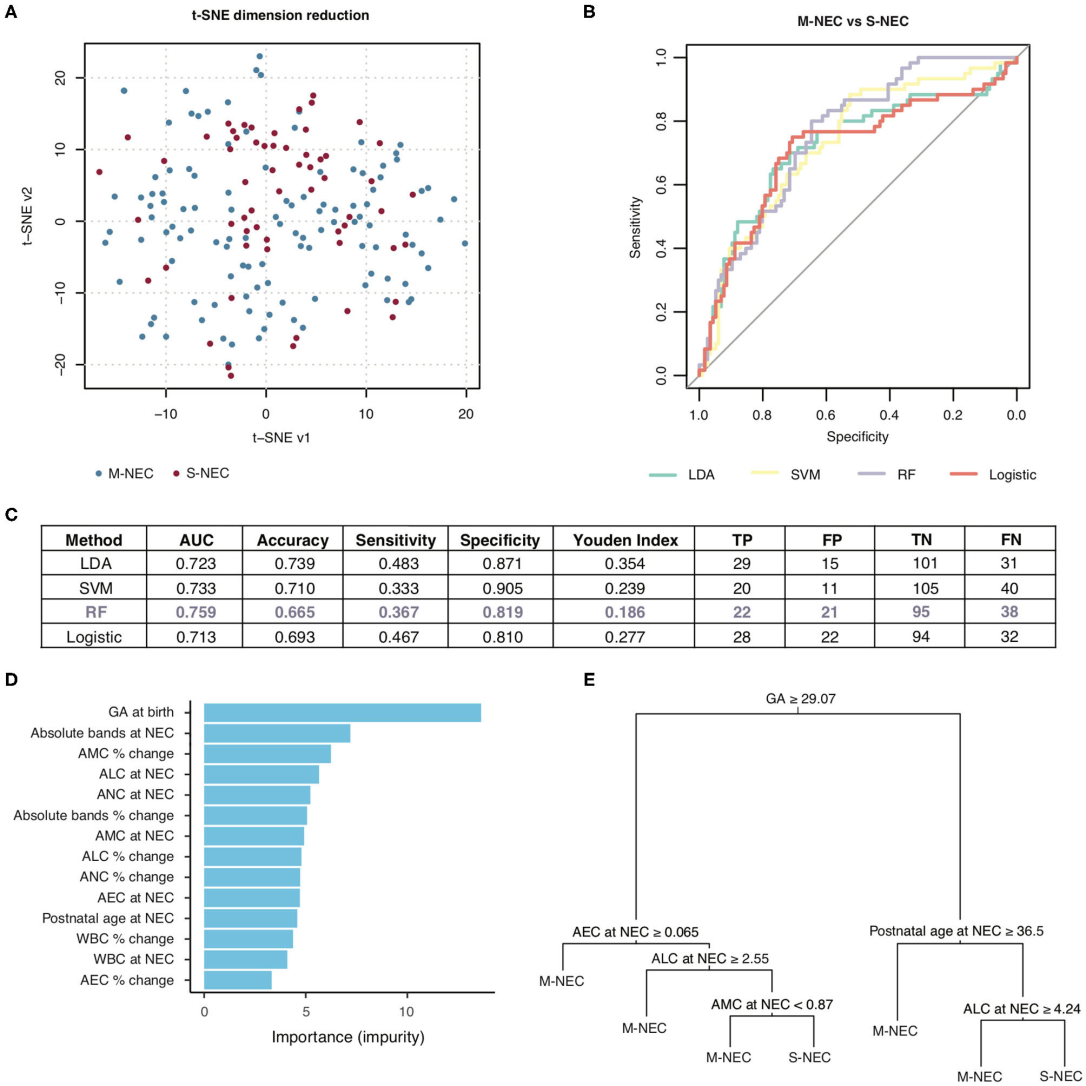
Machine learning prediction model for two-way comparison of S-NEC and medical NEC (M-NEC). **(A)** t-SNE separation of S-NEC and M-NEC. **(B)** Receiver operating characteristic curve for models using LDA, SVM, RF, and logistic regression. **(C)** Table of model characteristics. **(D)** Contribution of each CBC component to the model. **(E)** Representative decision tree. AUC, area under the curve; TP, true positive; FP, false positive; TN, true negative; FN, false negative.

Finally, we wanted to examine if predictive modeling using machine learning algorithms based on CBC values at the time of NEC diagnosis could distinguish between S-NEC, M-NEC, and controls. No separation using t-SNE could be established between the three groups (Supplementary Figure 5A). We were also unable to find a model that could predict which group the subjects belonged to based on their CBC features, using several different algorithms (Supplementary Figures 5B,C). However, in two-way comparisons, our models were able to accurately differentiate between S-NEC and controls (Figures 5A–E) and S-NEC and M-NEC (Figures 6A–E).

Of the models used for the comparison of S-NEC vs. controls, RF had the best performance with an area under the curve (AUC) of 0.877, sensitivity 0.8, and specificity 0.793, with the other models ranging from 0.777 to 0.802 for the AUC (Figures 5B,C). Absolute bands at NEC, ALC at NEC, and GA at birth were the top three contributors to the RF modeling, while AEC percent change contributed the least (Figure 5D). A representative decision tree with a cutoff for each component is shown in Figure 5E.

**Figure 7:**
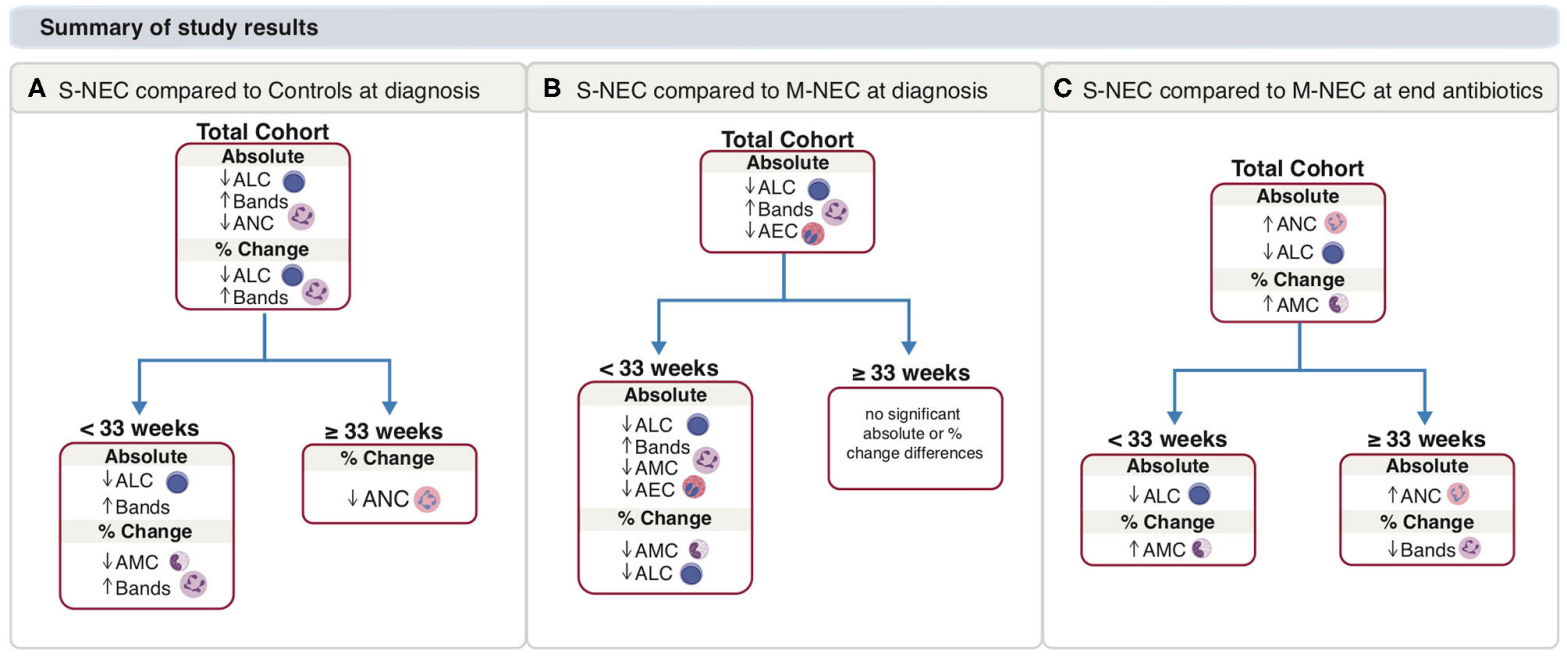
Main study results. Summary for S-NEC compared with controls **(A)** and M-NEC **(B)** for absolute and percent change differences at NEC diagnosis from baseline. **(C)** Summary for S-NEC compared with M-NEC for absolute and percent change differences at the end of antibiotics compared with NEC diagnosis.

For the two-way comparison of S-NEC vs. M-NEC, the AUC for the modeling was slightly worse than for the S-NEC vs. control comparison, with the RF modeling having the highest AUC of 0.759 and the others ranging between 0.713 and 0.733. Specificity was above 0.80 for all of the models (Figures 6B,C). However, sensitivity was low across the board (Figures 6B,C). GA at birth, absolute bands at NEC, and AMC percent change at NEC were the top three contributors to the RF modeling, while AEC percent change again contributed the least (Figure 6D). A representative decision tree with a cutoff for each component is shown in Figure 6E. A summary figure is shown in Figure 7.

## Discussion

Necrotizing enterocolitis is a devastating neonatal disease characterized by severe immune dysregulation, among other factors. While NEC occurs predominately in very pre-mature infants, late pre-term and term infants also develop NEC. Interestingly, there are suggestions that these groups have clinically and radiographically distinct disease presentations (25, 26). Adding to these distinctions, this study identified discrete signatures in circulating immune cells in a relatively large cohort of infants with NEC based on GA at birth. Moreover, we used predictive modeling tools to differentiate subjects with severe NEC who ultimately underwent surgery from control infants and infants with NEC who were treated medically based on CBC values obtained on day of NEC diagnosis. These models suggest that, together with clinical context in mind and the opinion of the medical and surgical teams, the CBC differential may be used to predict infants at high risk of requiring surgical intervention.

Compared with two previously published reports, the degree of change for monocytes at NEC onset in our study was smaller than previously reported (4, 5). In our cohort, illness onset occurred at earlier postnatal ages than prior studies investigating monocyte changes. Peripheral monocyte levels are known to vary over the first month of life in neonates (12, 14). Although we reported changes in monocytes from a normalized timepoint in an attempt to control for this fact, variability in our baseline measure may not fully correct for these fluctuations. Regardless, the acute drop in monocytes seen in our study is consistent with previous clinical and pre-clinical data that suggest recruitment of blood monocytes to the intestines during the acute phase of inflammation in NEC (27, 28).

Beyond changes in monocyte count, this study described the global immune response in NEC, as evident by changes in many peripheral cell types. Few other studies have evaluated changes in peripheral lymphocytes, neutrophils, bands, and eosinophils in infants with NEC; therefore, the results presented here provide valuable information about clinical laboratory data underlying NEC diagnosis and recovery. The few studies that have evaluated lymphocytes and mortality in NEC show conflicting results (9–11). While neonates with fulminant NEC were more likely to have lymphopenia (ALC <4.00 ×10^9^/L) at diagnosis (11), infants with NEC and lymphocytosis (>40% lymphocytes) at diagnosis had increased mortality compared with those without lymphocytosis (10). There may be a GA-based dichotomy underlying these data, as higher ALC was associated with increased mortality in younger GA infants, but decreased mortality in older GA infants (9). Indeed, these previously published cohorts differed in GA and BW distributions (10, 11). In the present study, we showed an acute drop in absolute lymphocytes at NEC onset in S-NEC compared with M-NEC and controls in the whole cohort, a relationship that appears to be driven by the group of infants <33 weeks. It is possible that lymphocyte recruitment and signaling in the intestines vary depending on pre-maturity and maturation of the immune system (29, 30). Proinflammatory T cells are recruited to the gut from peripheral blood in part due to TLR4 signaling in the intestines—signaling that is preferentially increased in pre-term compared with full-term intestinal tissue (29). Thus, the drop in lymphocytes seen in early pre-term infants described here may reflect this enhanced T cell recruitment to the gut from the peripheral circulation (29, 31).

Few recent studies have evaluated changes in neutrophils in NEC. However, results are largely congruent and suggest that neutropenia at NEC onset, a marker of systemic inflammation, is associated with increased severity and mortality (6–8, 32, 33). In the present study, we showed an acute decrease in peripheral neutrophils limited to late pre-term/term cases. Similarly, in a cohort composed primary of late pre-term infants with NEC, there was a similar association between neutropenia (ANC <1.5 ×10^9^/L) and worse outcomes (7). Deviations in neutrophil count and function are seen in SGA infants (34, 35), and there was a higher proportion of SGA infants within the older (≥33 weeks) subgroup of our cohort. Many have proposed hypoxia is the key trigger for NEC in both term and SGA infants (3, 36). Hypoxia induces neutrophil intestinal epithelial transmigration (37), and indeed, neutrophils are seen in intestinal tissue of infants with surgical NEC (27). Together, these data suggest the importance of neutrophil transmigration and hypoxia-driven intestinal recruitment in the development of NEC in late pre-term and term infants within our cohort. However, our results may also reflect a blunted neutrophil response in early pre-term infants due to a less developed immune system. Future studies are needed to characterize how neutrophils are recruited and migrate from the peripheral circulation to intestinal tissue in infants with NEC at varying levels of pre-maturity.

In addition to leukocyte patterns at NEC diagnosis, we evaluated the changes in peripheral cell counts in the recovery period from NEC, by analyzing CBCs obtained at the end of antibiotics for NEC cases. For infants <33 weeks, the differences in absolute monocyte count between S-NEC and M-NEC cases at diagnosis were no longer observed at the end of antibiotic treatment; however, S-NEC cases continued to have lower absolute lymphocyte count at the end of antibiotics, suggesting an incomplete lymphocyte recovery in S-NEC. Few studies have previously evaluated how peripheral immune cell populations change in the recovery process from NEC. In infants with NEC who undergo bowel resection, differences in the ratio of T cell populations within ileal tissue resolve between initial surgical resection and reanastomosis, suggesting that changes in immune cells during this recovery period are apparent at the tissue level (38). It is possible that lymphocyte recovery after NEC, as cells recruited to the gut migrate back from tissue to peripheral blood, occurs over a time period longer than the 10–15 days of antibiotics seen in the present study, but shorter than the time until reanastomosis. Further studies are needed in order to evaluate the effect of peripheral immune cells in infants with NEC during this recovery period, including in infants who develop recurrent NEC or post-NEC complications (e.g., stricture formation).

Through a comprehensive analysis of the CBC differential, we identified two discrete leukocyte signatures in S-NEC and M-NEC cases born <33 and ≥33 weeks. Together, these results suggest differences in peripheral immune cell responses in NEC that vary by pre-maturity and are apparent in a readily available CBCs. These hypothesis-generating results offer many future scientific and clinical directions, albeit beyond the scope of the present study. Whether these changes reflect immaturity of the immune system, immaturity of the intestinal system, or an evolving interplay between the two is not yet known. For instance, it is possible our observed changes in neutrophils limited to infants ≥33 weeks may reflect increased migration to intestinal tissue in late pre-term/term infants, or a blunted neutrophil migratory response due to less mature immune or adhesion systems in infants <33 weeks. Future mechanistic studies are needed in order to clearly delineate the relationship between the developing immune and intestinal systems in NEC and how these reflect clinical and laboratory data collected in real time.

Through machine learning algorithms, we were able to generate predictive models that may be able to predict which infants with NEC will require surgery. Indeed, these models used data from CBC differentials gathered on the day of NEC diagnosis, a median of 1 day prior to surgical intervention in infants with S-NEC. Although the most reliable model was able to predict S-NEC infants from controls with relatively high specificity and sensitivity, the models did not perform as well when differentiating S-NEC from M-NEC cases, with relatively low sensitivities and high numbers of false-positive S-NEC cases. Therefore, our models are likely unable to differentiate S-NEC and M-NEC when used in isolation, but may provide additional objective data before the decision to undergo surgery is finalized. In this way, our models may be used to help rule out S-NEC from M-NEC when the clinical course has yet to be determined.

A limitation of this study is the observed differences in postnatal ages at baseline and at NEC diagnosis between S-NEC, M-NEC, and controls. Within the entire cohort, control infants were significantly younger, in terms of chronological age, at diagnosis compared with both M-NEC and S-NEC. This difference was also seen when the cohort was stratified by GA <33 and ≥33 weeks. Peripheral cell counts are known to vary by postnatal age after birth in pre-term and term infants (12–14). We attempted to account for differences in postnatal age by using a normalized baseline timepoint as an internal control to compare against changes in peripheral cell counts at NEC diagnosis. Indeed, there were no observed differences in any peripheral cell count at baseline between S-NEC, M-NEC, and controls throughout our analysis, despite this postnatal age mismatch at baseline and NEC. This suggests that our baseline timepoint functioned as a normalized timepoint, and suggests that postnatal age differences are not the only contribution to the infants' CBC at these points. Furthermore, in our predictive models to differentiate S-NEC from both controls and M-NEC, postnatal age at diagnosis was not a top contributor to the overall modeling. Additionally, despite postnatal age differences at NEC, M-NEC and controls behaved similarly when compared with S-NEC in our study, especially for infants <33 weeks. Together, this suggests that the differences present at NEC diagnosis were more likely due to immune responses to NEC and not based solely on differences in postnatal age.

There are several other limitations of the present study. The first is the retrospective nature of the analysis. Because of this design, CBC values were not obtained at preset timepoints, but when clinically indicated. Although this introduces heterogeneity into the results, we believe this also makes the study more clinically applicable. In neonates, especially well-appearing or late pre-term infants, serial blood tests are limited. For this reason, most of the CBC values for our control infants were collected soon after birth and significantly earlier than in NEC cases, as discussed previously. Next, the S-NEC group within our cohort were younger gestationally and had more comorbidities than the other two groups, and we did not account for comorbidities in our analyses. Additionally, while the decision to take an infant to surgery is never taken lightly, there are no formal criteria for determining the need for surgery beyond evidence of free air on an abdominal x-ray, and therefore, this limits the standardization of which infants with NEC undergo surgery and when. Finally, there may be wide-ranging changes in peripheral counts at illness onset as cells transmigrate from blood to tissue, and this study was not able to detect such patterns.

## Conclusion

We identified two discrete immune patterns in NEC based on GA. For early pre-term infants (<33 weeks) with NEC, infants who ultimately required surgical intervention had a distinct leukocyte pattern from infants who were treated medically. In surgical NEC cases <33 weeks, NEC onset was characterized by an acute drop in monocytes and lymphocytes and a rise in band count compared with medical NEC. For late pre-term and term infants (≥33 weeks), medical and surgical NEC cases behaved similarly, and there was an acute drop in ANC at diagnosis for both medically and surgically treated cases. Together, these data help to better elucidate the CBC abnormalities that may underlie different mechanisms of this devastating disease in infants of all gestational ages, and support stratifying NEC based on pre-maturity.

## Data Availability Statement

The original contributions generated in the study are included in the article/Supplementary Material, further inquiries can be directed to the corresponding author.

## Ethics Statement

The studies involving human participants were reviewed and approved by University of Pittsburgh Institutional Review Board. Written informed consent to participate in this study was provided by the participants' legal guardian/next of kin.

## Author Contributions

JP conceptualized and designed the study, collected the data, carried out the initial analyses, drafted the initial manuscript, and revised the manuscript. SL performed predictive modeling analysis and helped to revise the manuscript. OO helped to conceptualize and design the study, participated in patient recruitment, and revised the manuscript. EP participated in data collection and reviewed the final manuscript. TY, MR, JB, BB, and MG identified and recruited patients for the study and contributed to and revised the final manuscript. LK supervised the entirety of the study including conceptualization, design, data collection, and writing of the manuscript. All authors approved the final manuscript as submitted and agreed to be accountable for all aspects of the work.

## Conflict of Interest

The authors declare that the research was conducted in the absence of any commercial or financial relationships that could be construed as a potential conflict of interest.
