# Supplementary material for: Gestational Age-Specific Complete Blood Count Signatures in Necrotizing Enterocolitis
Source: Front Pediatr. 2021 Feb 26;9:604899. doi: 10.3389/fped.2021.604899 (PMC7952609; doi:10.3389/fped.2021.604899)
Supplement: Supplementary file 1 [file Table_1.DOCX]

|  | **<33 weeks** | | | |  |  | | **≥33 weeks** | | | | |  |
| --- | --- | --- | --- | --- | --- | --- | --- | --- | --- | --- | --- | --- | --- |
|  | **Total**  **(n=187)** | **M-NEC**  **(n=81)** | **S-NEC**  **(n=56)** | **Controls**  **(n=50)** | ***p* value** | |  | | **Total**  **(n=59)** | **M-NEC**  **(n=35)** | **S-NEC**  **(n=5)** | **Controls**  **(n=19)** | ***p* value** |
| **Gestational Age,**  median (IQR), w+d | 29+3  (27+0 to 31+3) | 30+2  (28+1 to 31+5) | 27+2  (26+0 to 29+3) | 29+6  (28+0 to 31+4) | <.001^a^ | |  | | 34+1  (33+6 to 34+6) | 34+1  (33+4 to 35+2) | 34+3  (33+6 to 39+2) | 34+1  (34+0 to 34+6) | .89^a^ |
| **Birth Weight** |  |  |  |  |  | |  | |  |  |  |  |  |
| median (IQR), g | 1220 (863-1562) | 1390 (983-1673) | 884 (720-1182) | 1320 (1084-1678) | <.001^a^ | |  | | 2125 (1791-2570) | 2125 (1774-2634) | 2479 (1767-2695) | 2095 (1870-2435) | .89^a^ |
| >1500g, No. (%) | 54 (29%) | 31 (38%) | 7 (12%) | 16 (32%) | .004^b^ | |  | | 54 (92%) | 32 (91%) | 5 (100%) | 17 (90%) | .75^b^ |
| <1500g, No. (%) | 133 (71%) | 50 (62%) | 49 (88%) | 34 (68%) |  |  |  | | 5 (8.5%) | 3 (8.6%) | 0 (0%) | 2 (10%) |  |
| **Female,** No. (%) | 82 (43.9%) | 41 (51%) | 17 (30%) | 24 (48%) | .05^b^ | |  | | 22 (37%) | 10 (29%) | 2 (40%) | 10 (53%) | .22^b^ |
| **Comorbidities** |  |  |  |  |  | |  | |  |  |  |  |  |
| CLD, No. (%) | 53 (28.3%) | 18 (22%) | 28 (50%) | 7 (14%) | <.001^b^ | |  | | 2 (3.4%) | 2 (5.7%) | 0 (0%) | 0 (0%) | .49^b^ |
| IVH, No. (%) | 52 (27.8%) | 22 (27%) | 21 (38%) | 9 (18%) | .08^b^ | |  | | 7 (12%) | 5 (14%) | 1 (20%) | 1 (5.3%) | .52^b^ |
| PDA, No. (%) | 43 (23%) | 14 (17%) | 23 (41%) | 6 (12%) | <.001^b^ | |  | | 4 (6.8%) | 3 (8.6%) | 0 (0%) | 1 (5.3%) | .74^b^ |
| ROP, No. (%) | 55 (29.4%) | 26 (32%) | 23 (41%) | 6 (12%) | .004^b^ | |  | | 2 (3.4%) | 2 (5.7%) | 0 (0%) | 0 (0%) | .49^b^ |
| SGA, No. (%) | 12 (6.4%) | 4 (4.9%) | 6 (11%) | 2 (4%) | .29^b^ | |  | | 12 (20%) | 9 (26%) | 1 (20%) | 2 (10%) | .42^b^ |
| **Died,** No. (%) | 14 (7.5%) | 1 (1.2%) | 12 (21%) | 1 (2%) | <.001^b^ | |  | | 0 (0%) | 0 (0%) | 0 (0%) | 0 (0%) | >.99^b^ |
| **Postnatal age at NEC onset,** median (IQR), d | 11 (6-22) | 11 (7-20) | 13 (7-24) | 7 (1-19) | .03^a^ | |  | | 5 (2-9) | 7 (5-10) | 4 (2-5) | 1 (1-1) | <.001^a^ |
| **Postnatal age at baseline,** median (IQR), d | 1 (0-3) | 1 (0-3) | 1 (0-9) | 0 (0-1) | <.001^a^ | |  | | 1 (0-2) | 1 (0-4) | 1 (1-1) | 0 (0-1) | .005^a^ |
| **Time between baseline and NEC onset,** median (IQR), d | 7.5 (4-14) | 8 (5-15) | 7 (4-12) | 5 (1-14) | .11^a^ | |  | | 4 (1-6) | 5 (4-7) | 2 (1-4) | 1 (0-1) | <.001^a^ |
| **Postnatal age at end of antibiotics,** median (IQR), d | 25 (19-38) | 23 (17-30) | 34 (22-43) | --^d^ | .002^c^ | |  | | 18 (15-22) | 18 (15-22) | 17 (16-19) | --^d^ | .95^c^ |
| **Antibiotic duration,**  median (IQR), d | 12 (10-15) | 10 (9-13) | 15 (13-18) | --^d^ | <.001^c^ | |  | | 12 (9-14) | 10 (8-13) | 14 (13-15) | --^d^ | .03^c^ |
| **Positive blood culture at NEC diagnosis**, No. (%) | 13 (9.5%) | 2 (2.5%) | 11 (20%) | --^d^ | <.001^a^ | |  | | 2 (5%) | 0 (0%) | 2 (40%) | --^d^ | .01^a^ |

Chronic lung disease (CLD); intraventricular hemorrhage (IVH); patent ductus arteriosus (PDA); retinopathy of prematurity (ROP); small for gestational age (SGA); weeks (w); days (d); grams (g)

^a^Kruskal-Wallis test

^b^Fischer exact test

^c^Mann-Whitney

^d^Not applicable to control group
