# Supplementary material for: Gestational Age-Specific Complete Blood Count Signatures in Necrotizing Enterocolitis
Source: Front Pediatr. 2021 Feb 26;9:604899. doi: 10.3389/fped.2021.604899 (PMC7952609; doi:10.3389/fped.2021.604899)
Supplement: Supplementary file 2 [file Data_Sheet_1.PDF]

Figure S1. Peripheral cell counts in whole cohort

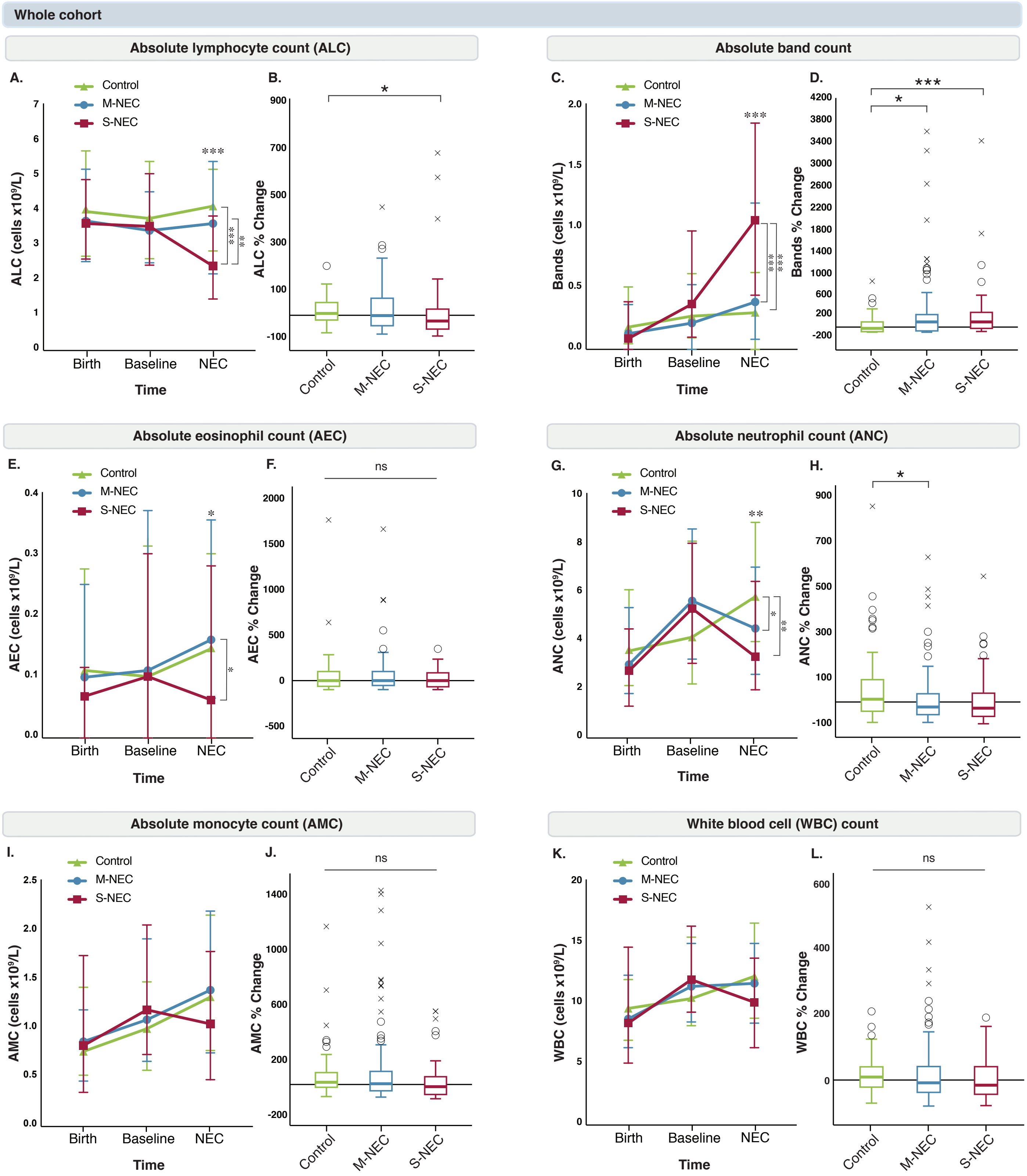

Figure S2. Peripheral cell counts in infants <33 weeks

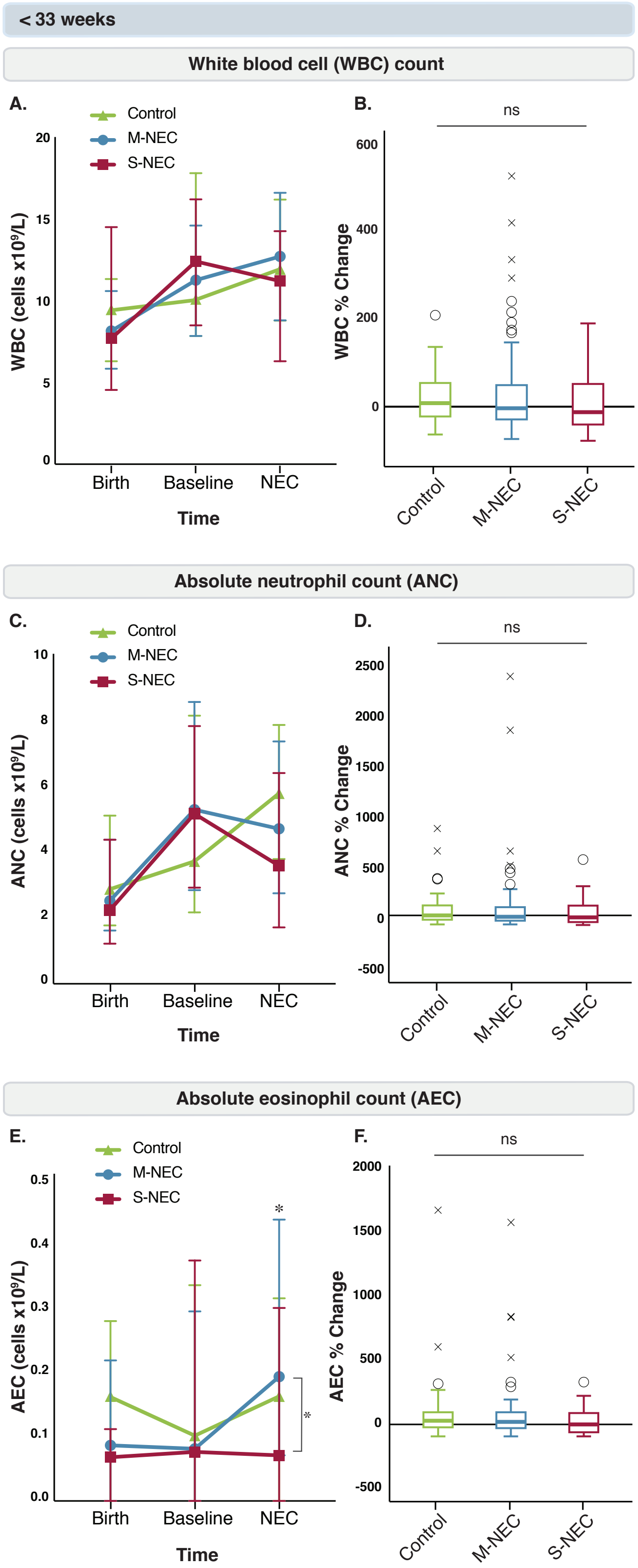

Figure S3. Peripheral cell counts in infants ≥33 weeks

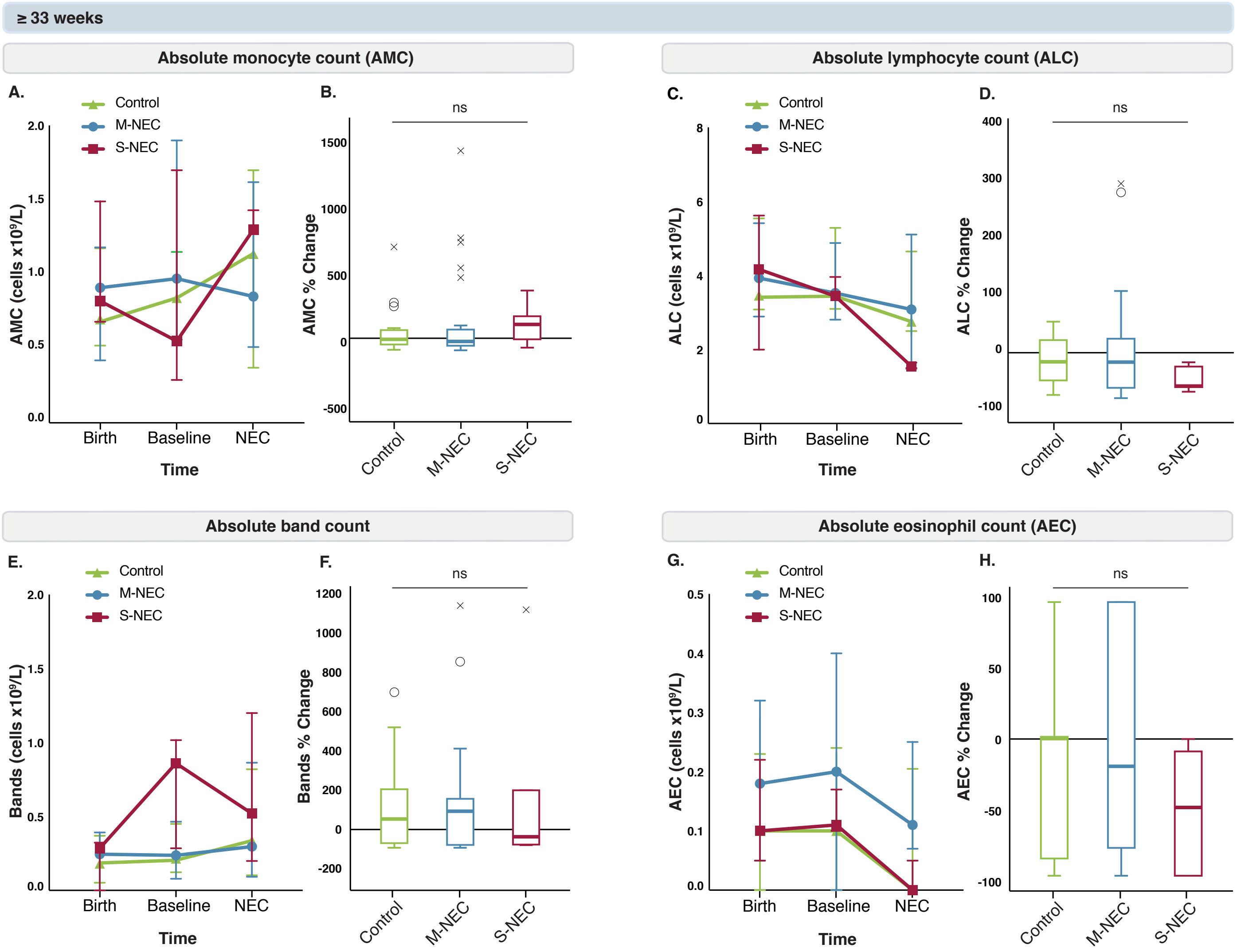

Figure S4. Peripheral cell counts at the end of antibiotics

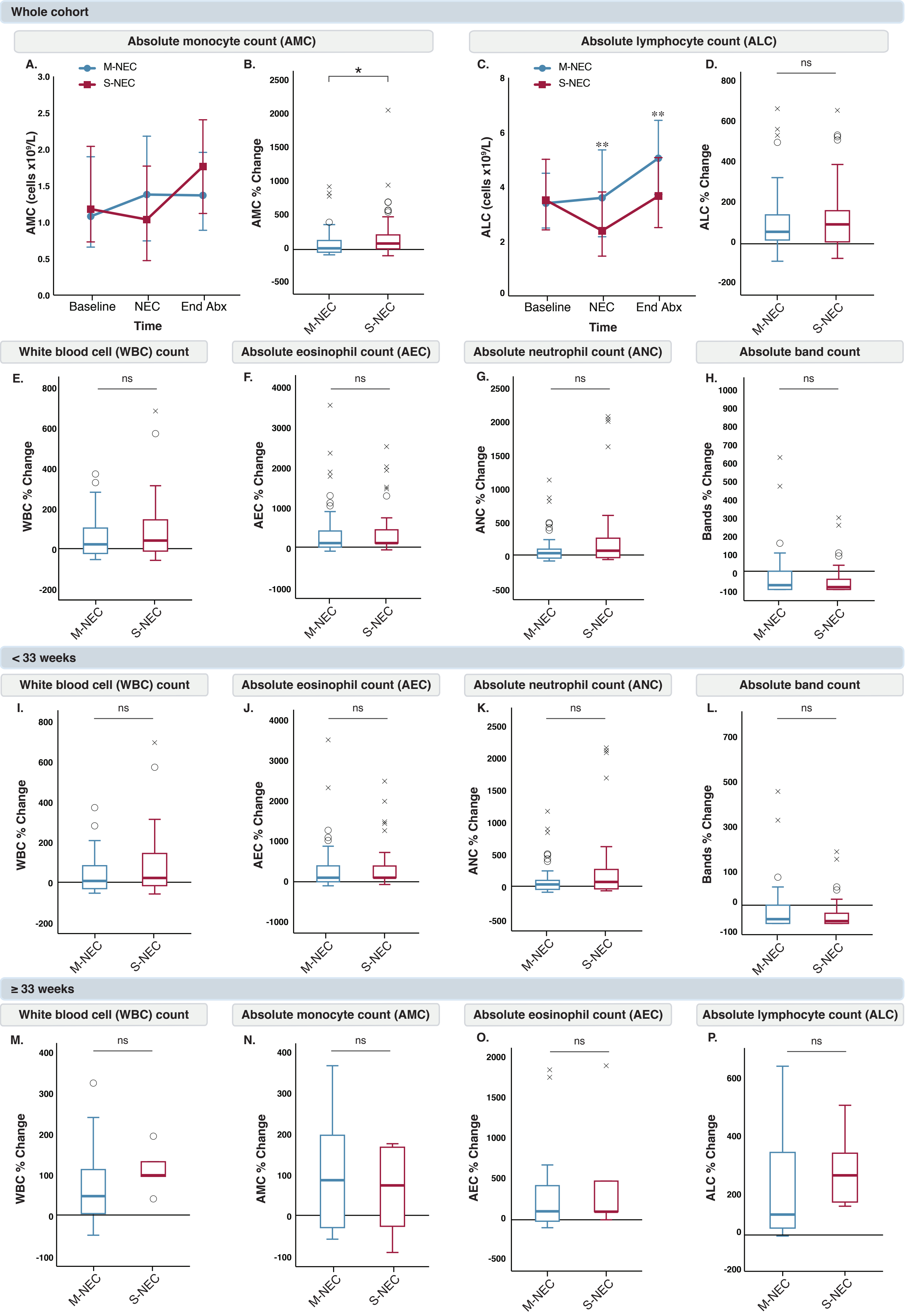

Figure S5. Predictive model for whole cohort

A.

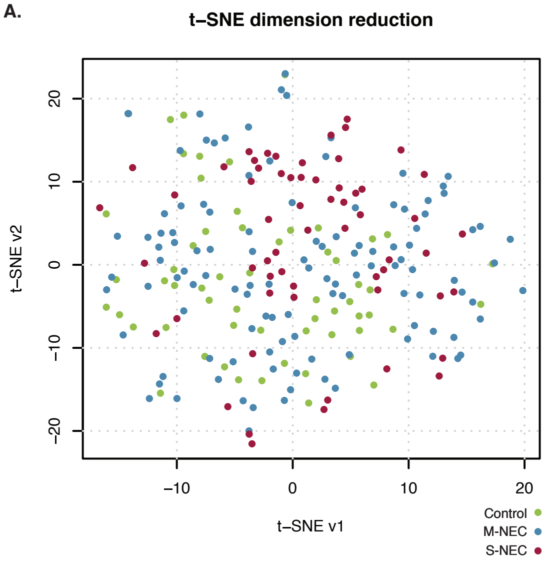

B.

LDA		Truth		
		Control	M-NEC	S-NEC
Prediction	Control	11	15	5
	M-NEC	41	83	25
	S-NEC	6	18	30

SVM		Truth		
		Control	M-NEC	S-NEC
Prediction	Control	7	4	3
	M-NEC	49	102	36
	S-NEC	2	10	21

RF		Truth		
		Control	M-NEC	S-NEC
Prediction	Control	16	9	1
	M-NEC	39	86	36
	S-NEC	3	21	23

C.

Method	Accuracy	ARI
LDA	0.529	0.076
SVM	0.556	0.078
RF	0.534	0.052
